# Systematic evaluation of monoclonal antibodies and immunoassays for the detection of Interferon-γ and Interleukin-2 in old and new world non-human primates

**DOI:** 10.1016/j.jim.2016.11.011

**Published:** 2016-11-24

**Authors:** Ankie Höglind, Irene Areström, Cecilia Ehrnfelt, Khosro Masjedi, Bartek Zuber, Luis Giavedoni, Niklas Ahlborg

**Affiliations:** a Mabtech, Box 1233, SE-131 28, Nacka Strand, Sweden; b Swedish Orphan Biovitrum AB, SE-112 76 Stockholm, Sweden; c Departments of Virology and Immunology and Southwest National Primate Research Center, Texas Biomedical Research Institute, San Antonio, TX 78245-0549, USA; d Department of Immunology, Stockholm University, SE-106 91 Stockholm, Sweden

**Keywords:** Antibody, ELISA, ELISpot, Interferon-γ, Interleukin-2, Non-human primates

## Abstract

Non-human primates (NHP) provide important animal models for studies on immune responses to infections and vaccines. When assessing cellular immunity in NHP, cytokines are almost exclusively analyzed utilizing cross-reactive anti-human antibodies. The functionality of antibodies has to be empirically established for each assay/application as well as NHP species.

A rational approach was employed to identify monoclonal antibodies (mAb) cross-reactive with many NHP species. Panels of new and established mAbs against human Interferon (IFN)-γ and Interleukin (IL)-2 were assessed for reactivity with eukaryotically expressed recombinant IFN-γ and IL-2, respectively, from Old (rhesus, cynomolgus and pigtail macaques, African green monkey, sooty mangabey and baboon) and New World NHP (Ma’s night monkey, squirrel monkey and common marmoset). Pan-reactive mAbs, recognizing cytokines from all NHP species, were further analyzed in capture assays and flow cytometry with NHP peripheral blood mononuclear cells (PBMC).

Pan-reactive mAb pairs for IFN-γ well as IL-2 were identified and used in ELISA to measure IFN-γ and IL-2, respectively, in Old and New World NHP PBMC supernatants. The same mAb pairs displayed high functionality in ELISpot and FluoroSpot for the measurement of antigen-specific IFN-γ and IL-2 responses using cynomolgus PBMC. Functionality of pan-reactive mAbs in flow cytometry was also verified with cynomolgus PBMC.

The development of well-defined immunoassays functional with a panel of NHP species facilitates improved analyses of cellular immunity and enables inclusion in multiplex cytokine assays intended for a variety of NHP.

## 1. Introduction

The similarity of the immune system of humans (HUM) and nonhuman primates (NHP) and the susceptibility of NHP to many human pathogens make NHP valuable animal models for studying immune responses to infectious diseases, preclinical evaluation of new therapeutics as well as for assessment of vaccine-induced immunity. Old World NHP including African green monkey (AGM), sooty mangabey (SM), macaques and baboons are used in studies on infection and vaccine development for many diseases (Grimaldi, 2008; Lin et al., 2009; Locher et al., 2001) with an important example being studies of acquired immunodeficiency syndrome (AIDS; Almond and Heeney, 1998; Hatziioannou et al., 2009). Simian immunodeficiency virus (SIV) infection in non-natural SIV hosts, such as rhesus (RHE), cynomolgus (CYN) and pigtail macaques (PTM), causes AIDS-like symptoms and provide models for assessment of vaccine-induced protective immunity (Sui et al., 2013). In natural SIV hosts like AGM and SM, SIV infection is less virulent and provides keys for the understanding of protective immune mechanisms induced by natural exposure (Sodora et al., 2009). Also New World NHP like common marmoset (MAR), squirrel monkey (SAI) and night monkeys, are susceptible to many HUM pathogens (Laws et al., 2013; Moncla et al., 2013) with an important area of research being *Plasmodium falciparum* and *P. vivax* malaria infection and evaluation of malaria vaccine development (Herrera et al., 2002; Jordan-Villegas et al., 2011).

Cellular immune responses induced by infection or vaccination are often assessed by analysis of cytokine production and hence monoclonal antibody (mAb) reagents reactive with cytokines from NHP are essential. Two important cytokines often analyzed as a measure of the activation state of the cell-mediated immune system are interferon (IFN) −γ and interleukin (IL)-2; cytokines that are produced primarily by CD4+ and CD8+ T cells and that are vital for the regulation and maintenance of both cellular and humoral immunity (Carter and Dutton, 1996). Important mAb-based capture assays used to quantify the levels of these cytokines as well as to enumerate the number of cytokine-secreting cells include ELISA and ELISpot, respectively. More recently, FluroSpot, originating from the ELISpot but utilizing fluorescent detection, has been developed to facilitate simultaneous analysis of e.g. IFN-γ and IL-2 at the single cell level (Gazagne et al., 2003). In addition to the capture assays based on mAb pairs for capture and detection, respectively, single mAbs reagents are used in applications like flow cytometry, immunohistochemistry, neutralization assays and western blot.

HUM and NHP cytokines are relatively conserved throughout evolution and mAbs to HUM cytokines often cross-react with the corresponding NHP protein. Hence studies in NHP almost exclusively utilize mAbs developed against human cytokines. The amino acid identity for most cytokines, including that of IFN-γ and IL-2, is around 95% between HUM and Old World NHP and around 90% between HUM and New World NHP. Despite the homology, cross-reactivity of mAb reagents has to be empirically established for each application/assay as well as for each species of NHP. Although the Old World NHP are evolutionary related as a group, as are the New World NHP, their cytokines generally differ at several residues between genera which may render mAbs cross-reactive with some, but not all, genera. Several NHP genera also include multiple species that are used in research and even between species within the same genus, cytokine sequences may differ (Hernandez et al., 2002; Tatsumi and Sata, 1997; Villinger et al., 1995).

The NHP cross-reactivity of many anti-HUM cytokine mAbs and capture assays is often defined when reagents are needed for a specific application in studies on a single NHP species; considering the large number of cytokines and immunological factors being analyzed, the many applications that mAbs are used for and the variety of NHP species used in research, this is a cumbersome way to gather information. In some studies, however, the cross-reactivity of mAb reagents and/or capture assays has been evaluated in NHP in a more systematic manner (Giavedoni, 2005; Kap et al., 2009; Makitalo et al., 2002; Riccio et al., 2015).

In the present study, a rational approach was taken to develop and evaluate mAb reagents and capture assays for detection of IFN-γ and IL-2 in both Old and New World NHP already at the stage when new mAbs to HUM IFN-γ and IL-2 were subjected to initial analyses. In addition, several previously established mAbs were included in the evaluation. Panels of mAbs were evaluated for cross-reactivity with NHP at an individual mAb level with eukaryotically expressed recombinant IFN-γ and IL-2 from various Old and New World NHP. MAbs displaying broad cross-reactivity were used to define single mAb reagents functional in flow cytometry and as capture/detection mAb pairs functional for all NHP species analyzed.

## 2. Materials and methods

### 2.1. Monoclonal antibodies to HUM IFN-γ and IL-2

MAbs to HUM IL-2 were made using methods previously described (Zuber et al., 2005). Briefly, BALB/c mice were immunized with *Escherichia coli*-derived recombinant HUM IL-2 (Peprotech, Rockville Hill, NJ, USA) and spleen cells were fused to Sp2/0 cells. Hybridoma supernatants were analyzed by ELISA for antigen reactivity. Strongly positive hybridomas were subcloned and mAb was purified from supernatants on Protein G columns. A part of the purified mAb was biotinylated. Animals were housed and handled at the Karolinska Institute, Solna, Sweden, according to the guidelines of the Swedish Ethical Committee for Animal Protection. Previously established mAbs to HUM IL-2 used were 17H12 and 249.9 (Mabtech, Nacka Strand, Sweden). The generation of mAbs to HUM IFN-γ is described in a recent study where the same mAbs were subjected to epitope mapping (Zuber et al., 2016). In addition, four previously established mAbs were included; 1-D1K, 7-B6-1, GZ4 and G23 (Mabtech). MAbs included in the analyses are listed in Table 1 as are isotype control mAbs with irrelevant specificity used in flow cytometry.

### 2.2. Eukaryotic expression of recombinant HUM and NHP IFN-γ and IL-2

Recombinant IFN-γ variants were made based on sequences obtained from Uniprot (http://www.uniprot.org) for the following species; HUM (*Homo sapiens*; Uniprot accession number P01579), RHE (*Macaca mulatta*; P63310), CYN, (*M. fascicularis*; G7PHZ5), PTM (*M. nemestrina*; P63311), olive baboon (BAB; *Papio hamadryas anubis*; Q865Y4), SM, (*Cercocebus atys*; P42162), Ma’s night monkey (AOT; *Aotus nancymaae*; O97542), MAR (*Callithrix jacchus*; B9VS86) and SAI (*Saimiri sciureus*; Q8MKF5). The sequence for AGM (*Chlorocebus sabaeus*) was provided by Francois Villinger, Emory University. All three macaque species have identical IFN-γ sequences. The last 12 amino acids in the C terminus of the IFN-γ sequence for AOT were not available and therefore the C terminus of MAR and SAI (SQTLFRGRRASQ) was added. A decapeptide tag designated BAM (DAEFRHDSGY) was added at the N terminus of all IFN-γ variants to enable detection of proteins with an anti-BAM mAb (Mabtech). Recombinant IL-2 was made based on Uniprot sequences from the same primates excluding AGM for which no sequence was available: HUM (P60568), RHE and PTM (P68290, P68291; identical sequences), CYN (Q29615), BAB (Q865Y1), SM (P46649), AOT (QJFM2), MAR (Q0PIT4) and SAI (Q8MKH2). The BAM tag was added at the C-terminus of all IL-2 variants. Plasmids (pIRES2-γFP1; Clontech, Mountain View, CA, USA) with synthesized genes encoding the respective cytokines with a leader sequence from mouse IgG kappa (METDTLLLWVLLLWVPGSTGD) and the BAM tag were made by GenScript (Piscataway, NJ, USA). The plasmid included a green fluorescent protein (GFP) reporter gene to assess transfection efficiency. Proteins were expressed in transfected human HEK cells as previously described (Arestrom et al., 2012) and HEK cell supernatants were used for specificity/reactivity analyses of antibodies.

### 2.3. HUM and NHP PBMC supernatants used for ELISA analysis

For the ELISA analysis of cytokine produced by HUM and NHP peripheral blood mononuclear cells (PBMC), blood was obtained from HUM and NHP in EDTA blood vials (BD, Franklin Lakes, NJ, USA) and PBMC were obtained using Ficoll separation as previously described (Giavedoni, 2005). In addition to HUM, PBMC were obtained from a yellow and olive baboon hybrid (*Papio hamadryas cynocephalus* and *P. h. anubis, respectively*; BAB*) as well as RHE, CYN, AGM, SM and MAR at the Southwest National Primate Research Center, San Antonio, TX, USA, in accordance with the regulations of the Texas Biomedical Research Institute Animal Care and Use Committee. HUM and NHP PBMC, at a concentration of 2 × 10^6^ cells/ml, were incubated at 37 °C and 5% CO_2_ in humidified air for 20 h. The cells were either incubated in medium only (unstimulated) or incubated with Staphylococcal enterotoxin type A and B (1 μg/ml; Sigma-Aldrich, St. Louis, MO, USA). Thereafter the supernatant was collected and frozen at −80 °C. After thawing, the supernatants were analyzed by capture ELISA.

### 2.4. HUM and CYN PBMC used for analysis in flow cytometry, ELISpot and FluoroSpot

CYN PBMC were obtained from animals handled and housed at the Primate Research Centre of the Swedish Institute for Infectious Disease Control, Stockholm, Sweden, in accordance with the guidelines of the Swedish Ethical Committee for Animal Protection. CYN PBMC were prepared as above. HUM PBMC were prepared as above from buffy coats obtained from anonymous blood donors (Blood Central, Karolinska University Hospital, Stockholm, Sweden). CYN and HUM PBMC were kept frozen and thawed prior to use as previously described (Dillenbeck et al., 2014).

### 2.5. Western blot and semi-quantitative determination of HUM and NHP IFN-γ and IL-2 concentrations in HEK cell supernatants

Transfected HEK cell supernatant containing HUM and NHP IFN-γ or IL-2 was added to NuPage LDS sample buffer (Invitrogen, Carlsbad, CA, USA) and kept at 70 °C for 10 min followed by being resolved under non-reducing conditions on NuPAGE 4–12% gradient Bis-Tris gels (Invitrogen) in a XCELL II Electrophoresis cell (Novex, San Diego, CA, USA) using NuPage MOPS running buffer (Invitrogen). A pre-stained standard (SeeBlue Plus 2; Invitrogen) was included as reference. The proteins were transferred in 20 mM Tris pH 8.6 to 0.2 μm pore size nitrocellulose membrane (Invitrogen) using a MiniTrans-Blot apparatus (Bio-Rad, Hercules, CA, USA). Membranes were blocked for 1 h at room temperature (RT) with 4% fetal calf serum (FCS) and 0.1% Tween 20 in phosphate-buffered saline (PBS). After washing with PBS, the membranes were incubated with 1 μg/ml of biotinylated mAb in PBS with 0.5% FCS. The membranes were washed and incubated for 1 h at RT with streptavidin-alkaline phosphatase (Mabtech) diluted 1:1000 in PBS, washed again and developed with BCIP/NBT Plus (Mabtech) for 5 min before being rinsed in tap water. To determine the concentration of NHP IFN-γ and IL-2, HUM IFN-γ and IL-2 in HEK supernatants was first quantified against recombinant standard cytokines by established cytokine ELISAs for HUM IFN-γ and IL-2, respectively, according to the instructions of the manufacturer (Mabtech). Following that, the relative amount of NHP IFN-γ and IL-2 in HEK cell supernatants was estimated by comparison to the HEK-derived HUM IFN-γ and IL-2 supernatant, respectively, using a biotinylated anti-BAM detection mAb and SA-ALP as above in Western blot. Western blot bands corresponding to IFN-γ and IL-2 from all species were analyzed by GelAnalyzer software (version 2010a; GelAnalyzer.com). The concentration of each NHP IFN-γ was then calculated by dividing the densitometric value obtained for a given NHP IFN-γ or IL-2 variant by the densitometric value obtained for HUM IFN-γ or IL-2 followed by multiplication of the HUM IFN-γ or IL-2 concentration determined by ELISA. The Western blot was repeated three times and the average concentration of each NHP IFN-γ and IL-2 variant was used. The functionality of anti-HUM IFN-γ and IL-2 mAbs in Western blot analysis was evaluated as above but using the antigen-specific mAbs for detection.

### 2.6. ELISA analysis of individual mAb reactivity with recombinant HUM and NHP IFN-γ and IL-2

Maxisorp 96-well plates (Nunc, Roskilde, Denmark) were coated at 4 °C overnight with 2 μg/ml of mAbs to IFN-γ or IL-2 in 100 μl PBS/well. Other assay steps were performed at RT. Five washes with 200 μl/well of PBS with 0.1% Tween 20 were made between all assay steps. After coating, wells were blocked for 1 h with incubation buffer (PBS with 0.05% Tween 20 and 0.1% bovine serum albumin; 200 μl/well). HEK supernatants containing IFN-γ or IL-2 with semi-quantified concentrations were added at serial dilutions in incubation buffer (100 μl/well) and incubated 2 h. Next, biotinylated anti-BAM tag mAb (Mabtech) at 1 μg/ml was added (100 μl/well) and incubated for 1 h. Following that, streptavidin-horse radish peroxidase conjugate (Mabtech) diluted 1:1000 in incubation buffer was added (100 μl/well) and incubated for 1 h at RT. The assay was developed with TMB substrate (Mabtech) and stopped with 0.18 M H_2_SO_4_ followed by absor-bance measurement at 450 nm–650 nm with an ELISA reader (Labsystems, Helsinki, Finland).

### 2.7. Capture ELISA analysis of HUM and NHP IFN-γ and IL-2

ELISA plates were coated as above with 2 μg/ml of mAbs to IFN-γ or IL-2. Blocking and washing between assay steps were performed as above. Recombinant HUM cytokine standards (Mabtech), HEK-derived supernatants with HUM or NHP IFN-γ or IL-2, or PBMC supernatant were added (100 μl/well) in serial dilutions in incubation buffer and incubated for 2 h at RT. Next, biotinylated mAbs against IFN-γ or IL-2 were added (100 μl/well) at 1 μg/ml and incubated for 1 h at RT followed by incubation of streptavidin-horseradish peroxidase, substrate development and analysis as above.

### 2.8. Flow cytometry

Transfected HEK cells expressing HUM or NHP IFN-γ or IL-2, as well as PBMC from HUM and CYN, either unstimulated or stimulated with PMA/Ionomycin cell stimulation cocktail (eBioscience, San Diego, CA, USA), and treated with Brefeldin A (Becton Dickinson, Franklin Lakes, NJ, USA), were fixed and permeabilized using BD Cytofix/Cytoperm kit (Becton Dickinson). Transfected HEK cells were stained with anti-IFN-γ, anti-IL-2 or isotype/subclass-matched control mAbs at 2 μg/ml for 30 min at 4 °C. Staining was detected using PE-conjugated F(ab)2 fragment goat anti-rat IgG or F(ab)2 fragment goat anti-mouse IgG (both diluted 1:50; Jackon Immunoresearch Inc., West Grove, PA, USA) for 30 min at 4 °C in the dark. HUM and CYN PBMC were stained with anti-IFN-γ (MT126L) and anti-IL-2 (MT8G10-biotin) at 2 μg/ml for 30 min at 4 °C, which was detected with Alexa Fluor® 488-conjugated F(ab)2 fragment mouse anti-rat (diluted 1:50; Jackon Immunoresearch Inc.) and SA-PE (3 μg/ml; Becton Dickinson) for 30 min at 4 °C in the dark. Cells were acquired in a Guava EasyCyte Mini flow cytometer (Millipore, Billerica, MA, USA). Data was analyzed using the FlowJo software 7.6.5 (FlowJo, Ashland, OR, USA).

### 2.9. ELISpot for HUM and CYN IFN-γ and IL-2 PBMC

ELISpot was performed essentially as described (Dillenbeck et al., 2014) using EtOH-activated polyvinylidene fluoride 96-well plates (Mabtech) coated with 100 μl of mAbs to IFN-γ or IL-2 at 15 μg/ml. HUM and CYN PBMC at 2.5 × 10^5^ cells/well with or without serial dilutions of cell lysate from HEK cells expressing RHE cytomegalovirus (CMV) pp65 in 100 μl cell culture medium were added. Recombinant pp65 was produced in the same way as the NHP cytokines, but without any tag, based on the Uniprot sequence Q772N2-RHCM6-Rh112. Despite the signal peptide, pp65 was poorly secreted and cell lysates were instead used for stimulation of PBMC. HEK cell lysates were made using M-PER buffer (Thermo Scientific, Stockholm, Sweden) according to the manufacturer’s instructions. The optimal dilution of cell lysate for stimulation was determined in pilot tests with serial dilutions of cell lysate (data not shown). As a positive control, 0.5 × 10^5^ PBMC/well stimulated with 0.1 μg/ml anti-CD3 mAb (Mabtech) was used. PBMC were incubated at 37 °C and 5% CO_2_ in humidified air for 20 h. After incubation, the plates were washed in PBS and incubated with biotinylated detection mAbs to IFN-γ or IL-2 (1 μg/ml in 100 μl PBS/0.5% FBS) at RT for 2 h, followed by incubation with streptavidin-horseradish peroxidase and subsequently TMB substrate (Mabtech). ELISpot assays were set up in triplicates. An iSpot Spectrum reader (AID, Strassberg, Germany) was used to enumerate the spots representing cytokine-producing cells.

### 2.10. FluoroSpot for simultaneous detection of CYN IFN-γ and IL-2

FluoroSpot was performed essentially as described (Dillenbeck et al., 2014) using EtOH-activated low fluorescent polyvinylidene fluoride 96-well plates (Millipore) coated with 100 μl of mAbs to IFN-γ and IL-2 at 15 μg/ml each. HUM and CYN PBMC were incubated as described in Section 2.9. After incubation, the plates were washed in PBS and incubated with biotinylated detection mAb to IL-2 and FITC-labeled detection mAb to IFN-γ (1 μg/ml of each mAb in 100 μl PBS/0.5% FBS) at RT for 2 h. After that, streptavidin (diluted 1:200) and anti-FITC mAb (diluted 1:200) labelled with fluorophores absorbing/emitting light at 490/520 and 550/570 nm (Mabtech), respectively, were incubated for 1 h. An iSpot Spectrum reader (AID) was used to analyze the green and red spots representing IFN-γ- and IL-2-producing cells, respectively. Spots representing double-producing cells were identified by their position on a computerized image overlay analysis.

## 3. Results

### 3.1. Individual reactivity of mAbs with recombinant HUM and NHP IFN-γ and IL-2

A panel of new mAbs made against HUM IFN-γ (*n* = 24) and IL-2 (*n* = 19) were analyzed for reactivity with recombinant HUM IFN-γ and IL-2, respectively (data not shown). Of these, eight mAbs to IFN-γ and seven mAbs to IL-2 that displayed the strongest reactivity with human cytokines were analyzed by ELISA for their ability to capture human and NHP IFN-γ and IL-2 expressed in transfected HEK cells; in addition, four previously established mAbs to human IFN-γ and two against human IL-2 were included in the analysis (Table 1).

**Fig. 1:**
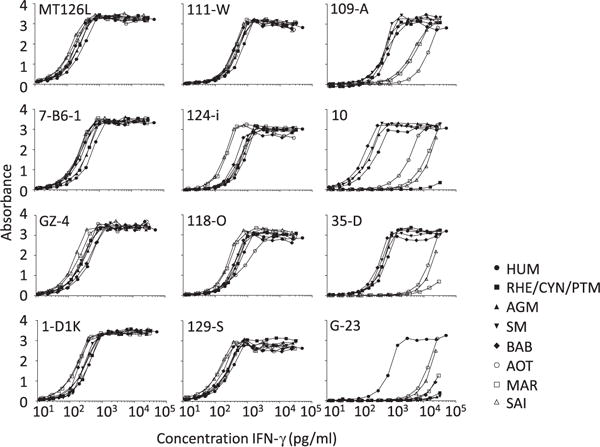
ELISA analysis of individual monoclonal antibodies (mAbs) to human (HUM) IFN-γ for cross-reactivity with non-human primate (NHP) IFN-γ. Anti-HUM IFN-γ mAbs were coated in ELISA plates and allowed to bind serial dilutions of HUM and NHP IFN-γ. The ability of the mAbs to capture the different IFN-γ variants was detected using a mAb to the peptide tag BAM in the N terminus of IFN-γ. The symbols correspond to IFN-γ from HUM, rhesus (RHE), cynomolgus (CYN) and pigtail macaque (PTM; all three macaques have identical IFN-γ sequences) as well as African green monkey (AGM), sooty mangabey (SM), olive baboon (BAB), Ma’s night monkey (AOT), common marmoset (MAR) and squirrel monkey (SAI). All samples were tested in duplicates and shown is the absorbance value. The experiment was repeated with reproducible results.

Eight mAbs (MT126L, 7-B6-1, GZ-4, 1-D1K, 111-W, 124-i, 118-O and 129-S) to HUM IFN-γ displayed a strong and comparable reactivity with IFN-γ from all species tested (Fig. 1). Two other mAbs (109-A and 35-D) displayed comparable reactivity with HUM IFN-γ and Old World NHP IFN-γ but low reactivity with New World NHP IFN-γ; mAb 10 displayed a similar pattern but was completely devoid of reactivity with macaque IFN-γ. MAb G23 displayed reactivity with HUM IFN-γ but poor reactivity with NHP IFN-γ.

**Fig. 2:**
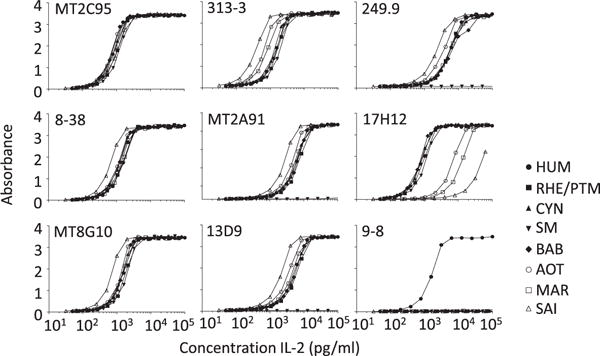
ELISA analysis of individual monoclonal antibodies (mAbs) to human (HUM) IL-2 for cross-reactivity with non-human primate (NHP) IL-2. Anti-HUM IL-2 mAbs were coated in ELISA plates and allowed to bind serial dilutions of HUM and NHP IL-2. The ability of the mAbs to capture the different IL-2 variants was detected using a mAb to the peptide tag BAM in the C terminus of IL-2. The symbols correspond to IL-2 from HUM, rhesus (RHE) and pigtail macaque (PTM; RHE and PTM have identical IL-2 sequences) as well as cynomolgus macaque (CYN), sooty mangabey (SM), olive baboon (BAB), Ma’s night monkey (AOT), common marmoset (MAR) and squirrel monkey (SAI). All samples were tested in duplicates and shown is the absorbance value. The experiment was repeated with reproducible results.

Four of the mAbs to IL-2 (MT2C95, 8-38, MT8G10 and 313-3) displayed a comparable reactivity with IL-2 from all species (Fig. 2). Three mAbs (MT2A91, 13D9 and 249.9) reacted equally well with all NHP except that they were completely devoid of reactivity with SM IL-2. MAb 17H12 reacted well with HUM and Old World NHP IL-2, but had a reduced reactivity with New World NHP IL-2; mAb 9-8 only recognized HUM IL-2.

### 3.2. Flow cytometry analysis of intracellular HUM and NHP IFN-γ and IL-2

**Fig. 3:**
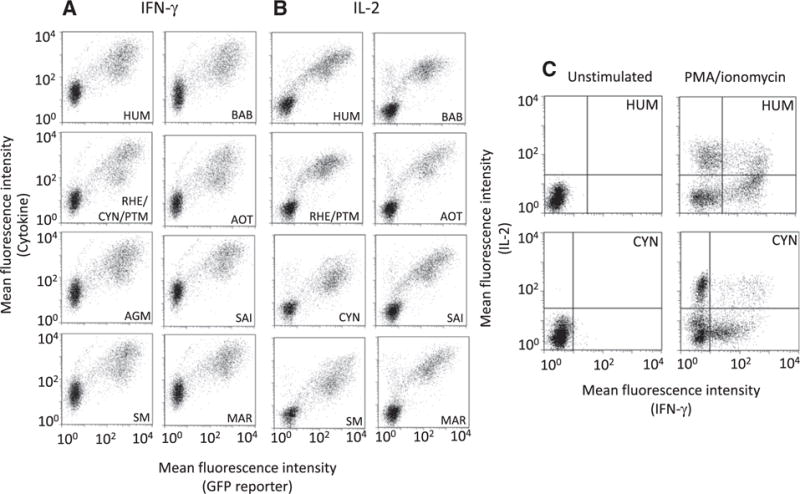
Flow cytometry analysis of the functionality of monoclonal antibodies (mAb) to human (HUM) IFN-γ and IL-2 with HEK cells secreting non-human primate (NHP) IFN-γ and IL-2. A) HEK cells expressing HUM and NHP IFN-γ were stained with anti-HUM mAb MT126L followed by goat anti-rat IgG-PE and the fluorescence intensity is shown on the Y-axis. The graphs show staining with HEK cells transfected with plasmids encoding IFN-γ from HUM, rhesus (RHE), cynomolgus (CYN) and pigtail macaque (PTM; all three macaques have identical IFN-γ sequences) as well as African green monkey (AGM), sooty mangabey (SM), olive baboon (BAB), Ma’s night monkey (AOT), squirrel monkey (SAI), and common marmoset (MAR). The degree of transfection efficiency is indicated by the green fluorescence protein (GFP) reporter shown on the X-axis. Staining with isotype controls was only positive in the GFP channel (data not shown). B) HEK cells expressing HUM and NHP IL-2 were stained with anti-HUM mAb MT8G10 followed by goat anti-mouse IgG-PE and the fluorescence intensity is shown on the Y-axis. The degree of transfection efficiency is indicated by the GFP reporter and is shown on the X-axis. The graphs show staining with HEK cells transfected with plasmids encoding IL-2 from HUM, RHE and PTM (RHE and PTM have identical IL-2 sequences) as well as CYN, SM, BAB, AOT, SAI and MAR. Staining with isotype controls was only positive in the GFP channel (data not shown). C) HUM and CYN peripheral blood mononuclear cells, either unstimulated or stimulated with PMA/Ionomycin, were stained with MT126L (IFN-γ) and MT8G10-biotin (IL-2) followed by goat anti-rat IgG-Alexa Fluor® 488 and streptavidin-PE. Fluorescence intensity for staining of IFN-γ is shown on the X-axis and staining of IL-2 is shown on the Y-axis. The experiments were repeated with reproducible results.

All mAbs were analyzed in flow cytometry with transfected HEK cells expressing HUM IFN-γ or IL-2 (data not shown); selected mAbs were further analyzed for their functionality with HEK cells expressing NHP cytokines; MAb MT126L displayed the best reactivity with IFN-γ (Fig. 3A) and mAb MT8G10 with IL-2 (Fig. 3B); these mAbs reacted equally well with HUM and NHP IFN-γ and IL-2, respectively. The signal intensity of the GFP reporter indicated that transfected HEK cells expressed all IFN-γ and IL-2 variants at comparable levels. The functionality of MT126L and MT8G10 with endogenous cytokines was verified using HUM and CYN PBMC stimulated with PMA/Ionomycin. Staining of IFN-γ, IL-2 and double-positive cells was comparable between HUM and CYN cells (Fig. 3C).

### 3.3. MAb reactivity with recombinant HUM and NHP IFN-γ and IL-2 in western blot

**Fig. 4:**
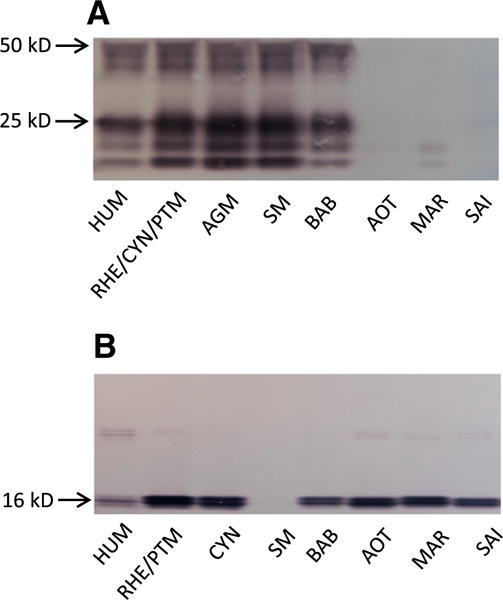
Western blot analysis of mAb reactivity with human (HUM) and non-human primate (NHP) IFN-γ and IL-2. Supernatants from HEK cells expressing HUM and NHP IFN-γ (approximately 15 ng/lane) were separated on a SDS-PAGE gel and transferred to a Western blot membrane. The cytokines were detected using mAbs to the respective cytokines. A) Detection of IFN-γ using mAb 111W. The image shows detection of IFN-γ derived from HUM, rhesus (RHE), cynomolgus (CYN) and pigtail macaque (PTM; all three macaques have identical IFN-γ sequences) as well as African green monkey (AGM), sooty mangabey (SM), olive baboon (BAB), Ma’s night monkey (AOT), squirrel monkey (SAI), and common marmoset (MAR). The molecular weight of fully glycosylated dimers (50 kD) as well as monomers (25 kD) are indicated by arrows. Below these bands are bands corresponding to dimers and monomers, respectively, with incomplete glycosylation. B) Detection of IL-2 using mAb 13D9. The image shows detection of IL-2 derived from HUM, RHE and PTM (RHE and PTM have identical IL-2 sequences) as well as CYN, SM, BAB, AOT, SAI and MAR. The molecular weight of IL-2 (16 kD) is indicated by an arrow. The experiments were repeated with reproducible results.

All mAbs were analyzed for reactivity with recombinant HUM IFN-γ or IL-2 in western blot. For IFN-γ, only mAb 111W displayed a strong signal and 7-B6-1 and G23 weaker signals (data not shown). The other mAbs were not reactive. MAb 111W was then analyzed for reactivity with recombinant HUM and NHP IFN-γ and stained IFN-γ from HUM and Old World NHP well but, in sharp contrast to the individual cross-reactivity analysis of mAb 111W in ELISA (Fig. 1), no or very little reactivity with New World NHP IFN-γ was seen in Western blot (Fig. 4A). The IFN-γ bands observed represent fully glycosylated monomers (around 25 kD) and dimers (around 50 kD) as well monomers and dimers with incomplete glycosylation as has been observed before with eukaryotically expressed HUM IFN-γ (Arakawa et al., 1986). For IL-2, several mAbs were functional; mAb 13D9, MT2A91 and 249 yielded the strongest bands (only 13D9 shown in Fig. 4B). These mAbs detected IL-2 from all species equally well apart from SM IL-2, in line with the ELISA single mAb reactivity analysis of the mAbs (Fig. 2).

### 3.4. MAb pair reactivity with recombinant HUM and NHP IFN-γ and IL-2 by capture ELISA

**Fig. 5:**
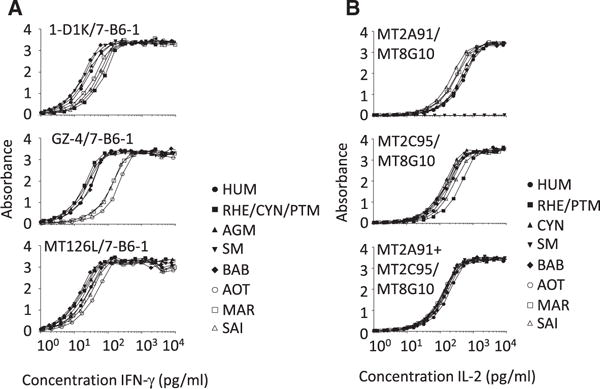
Reactivity with non-human primate (NHP) IFN-γ and IL-2 displayed by capture ELISA systems based on monoclonal antibodies (mAb) to human (HUM) IFN-γ and IL-2. A) For IFN-γ, three different capture mAbs, 1-D1K, GZ-4 and MT126L, were evaluated in combination with the biotinylated detection mAb 7-B6-1 for reactivity with serial dilutions of HUM and NHP IFN-γ. IFN-γ variants tested were from HUM, rhesus (RHE), cynomolgus (CYN) and pigtail macaque (PTM; all three macaques have identical IFN-γ sequences) as well as African green monkey (AGM), sooty mangabey (SM), olive baboon (BAB), Ma’s night monkey (AOT), squirrel monkey (SAI), and common marmoset (MAR). B) For IL-2, two different capture mAbs, MT2A91 and MT2C95, were used alone or as a mixture and in combination with the biotinylated detection mAb MT8G10 for reactivity with serial dilutions of HUM and NHP IL-2. IL-2 variants tested were from HUM, RHE and PTM (RHE and PTM have identical IL-2 sequences) as well as CYN, SM, BAB, AOT, SAI and MAR. All samples were tested in duplicates and shown is the absorbance value. The experiment was repeated with reproducible results.

All mAbs to HUM IFN-γ were tested in all possible capture and detection mAb combinations with HUM IFN-γ and the most sensitive combinations were further analyzed for reactivity with NHP IFN-γ (data not shown). The three systems displaying the highest reactivity with NHP IFN-γ all included mAb 7-B6-1-biotin for detection, combined with either of three different capture mAbs, 1-D1K, GZ4 or MT126L (Fig. 5A). MAbs 1-D1K/7-B6-1-biotin detected HUM and Old World NHP IFN-γ comparably well with the exception of macaque IFN-γ that was slightly less well recognized as was New World NHP IFN-γ. MAbs GZ-4/7-B6-1-biotin, on the other hand, displayed comparable reactivity with HUM and all Old World NHP but lower reactivity with New World NHP. This contrasted to the ELISA analysis of individual mAb reactivity with HUM and NHP IFN-γ where GZ4 as well as 7-B6–1 displayed high reactivity with Old and New World NHP (Fig. 1). MT126L/7-B6-1-biotin displayed high reactivity with all species. The three mAb combinations were also compared for reactivity with IFN-γ from PHA-stimulated CYN and MAR PBMC which confirmed that MT126L/7-B6-1 displayed higher reactivity with both species compared to the other mAb combinations (data not shown).

In the same manner, anti-HUM IL-2 mAb combinations were tested in capture ELISA for reactivity with HUM IL-2 and a large number of functional combinations were identified and further analyzed for reactivity with NHP IL-2 (data not shown). The most sensitive systems with high reactivity to NHP IL-2 included either capture mAb MT2A91 or MT2C95 combined with the detection mAb MT8G10 (Fig. 5B). However, the system including MT2A91 did not detect SM IL-2, in line with results obtained with the ELISA analysis of individual mAbs (Fig. 2). The system using MT2C95 for capture displayed a comparable reactivity with all NHP except for a slightly lower reactivity with RHE/PTM IL-2. To overcome the lower reactivity with certain species, the two capture mAbs were used together as a mixture which resulted in a comparable reactivity with IL-2 from all species (Fig. 5B).

### 3.5. Capture ELISA reactivity with IFN-γ and IL-2 in HUM and NHP PBMC supernatants

**Fig. 6:**
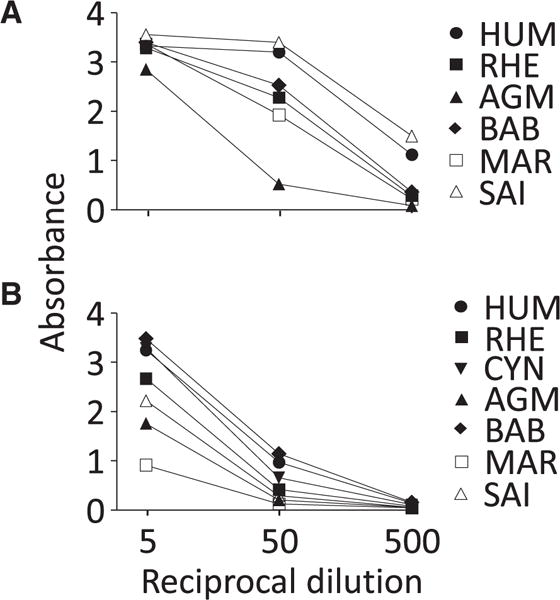
Reactivity of capture ELISAs with IFN-γ and IL-2 from stimulated peripheral blood mononuclear cells (PBMC) from human (HUM) and non-human primates (NHP). Supernatants from PBMC stimulated with SEA/B were analyzed by capture ELISAs for IFN-γ (A) and IL-2 (B). A) IFN-γ was measured using mAb MT126L/7-B6-1-biotin in PBMC supernatants from human (HUM), rhesus macaques (RHE; identical IFN-γ sequence with pigtail (PTM) and cynomolgus (CYN) macaque), African green monkey (AGM), baboon (BAB*; yellow and olive baboon hybrid), common marmoset (MAR) and squirrel monkey (SAI). B) IL-2 was measured using mAb MT2A91 + MT2C95/MT8G10-biotin in PBMC supernatants from HUM, RHE (identical IL-2 sequence with PTM), CYN, AGM, BAB*, MAR and SAI. Supernatants were diluted 5, 50 and 500 times before analysis. All samples were tested in duplicates and shown is the absorbance value. The results of the IFN-γ ELISA was confirmed with other PBMC supernatants from the same NHP whereas the IL-2 ELISA was performed once.

To verify the reactivity established with recombinant IFN-γ, the capture ELISA based on MT126L/7-B6-1-biotin was used to measure IFN-γ in superantigen-stimulated PBMC supernatants from HUM, RHE (identical IFN-γ sequence to CYN and PTM), AGM, BAB*, MAR and SAI. IFN-γ from all species was well recognized (Fig. 6A). When quantifying the supernatants against a recombinant HUM IFN-γ standard, the highest levels were found in SAI supernatant (49 ng/ml) and the lowest in AGM supernatant (1.7 ng/ml). In unstimulated PBMC supernatants, IFN-γ was undetectable except for AGM and BAB* supernatants that had levels of 68 and 187 pg/ml, respectively (data not shown). It should be noted that PBMC were from one individual per species and hence differences between species is likely to be due to individual differences in PBMC responsiveness rather than reflecting different degrees of species cross-reactivity displayed by the ELISA. Potentially, differences between species in responsiveness to the superantigen stimuli used may also have an impact on the levels measured.

In a similar manner, the capture ELISA for IL-2 based on MT2A91 + MT2C95/MT8G10-biotin was used to measure IL-2 in superantigen-stimulated PBMC supernatants from HUM, RHE (identical IL-2 sequence to PTM), CYN, AGM, BAB*, MAR and SAI. IL-2 from all species was well recognized (Fig. 6B); AGM IL-2, due to the lack of an available amino acid sequence, was not included in the analyses with recombinant IL-2 but was here shown to be recognized by the human IL-2 capture mAb system. The highest level was found in BAB supernatant (4.4 ng/ml) and the lowest in MAR supernatant (0.36 ng/ml); IL-2 levels in supernatant from unstimulated PBMC were below 10 pg/ml (data not shown). Similar to IFN-γ, differences in IL-2 levels are likely to be due to individual PBMC responsiveness.

### 3.6. Evaluation of capture systems for CYN IFN-γ and IL-2 in ELISpot and FluoroSpot

**Fig. 7:**
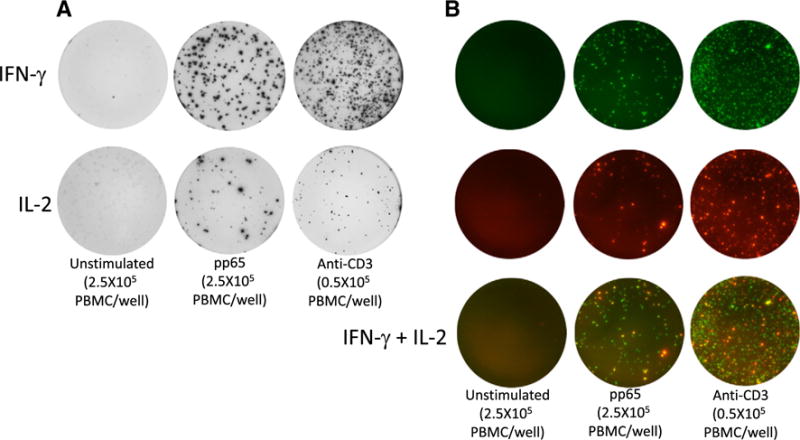
ELISpot and FluoroSpot analysis of IFN-γ and IL-2 secreted by peripheral blood mononuclear cells (PBMC) from cynomolgus macaque. PBMC were incubated overnight in medium only or stimulated with rhesus cytomegalovirus pp65 or anti-CD3 mAb. A) ELISpot analysis of IFN-γ responses using the mAb combination MT126L and 7-B6-1-biotin and IL-2 using the mAb combination MTA91/MT2C95 and MT8G10-biotin. B) FluoroSpot analysis of IFN-γ and IL-2 using the same antibodies as in ELISpot but in the same well. Images shown represent IFN-γ responses analyzed with a reader filter for fluorophores absorbing and emitting light at 490/550 and IL-2 responses analyzed with a reader filter for 550/570 nm. Shown is also a computerized overlay analysis of the IFN-γ and IL-2 images (IFN-γ + IL-2) where double-producing cells are visualized as yellow spots. The experiments were repeated with reproducible results.

The optimal mAb combinations for detection of IFN-γ and IL-2 in Old and New World NHP species defined by ELISA, were evaluated in ELISpot using CYN PBMC stimulated with recombinant pp65 from RHE CMV; most macaques in captivity are infected with RHE CMV and can be expected to have a cellular immune response to pp65. Antigen-specific responses against pp65 manifested both by IFN-γ and IL-2 production were observed, with more cells responding by IFN-γ secretion (Fig. 7A). The same experiment was performed using FluoroSpot where IFN-γ and IL-2 can be detected simultaneously in the same well (Fig. 7B). Similar results as in the ELISpot were obtained with a triplicate average of 156 IFN-γ spots and 54 IL-2 spots in response to pp65. By a computerized overlay analysis of IFN-γ and IL-2 images, based on the position of IFN-γ and IL-2 spots, 94% of all IL-2 spots were defined as being derived from cells secreting both IFN-γ and IL-2. HUM PBMC tested in parallel yielded comparable polyclonal responses after stimulation with anti-CD3 mAb but did not respond to RHE CMV pp65 (data not shown).

## 4. Discussion

Panels of mAbs to human IFN-γ or IL-2 were analyzed for cross-reactivity with cytokines derived from Old and New World NHP species in order to identify functional single mAb reagents and mAb-based capture immunoassays. MAbs for flow cytometry and mAb pairs for ELISA, ELISpot and Fluorospot, displaying reactivity with IFN-γ or IL-2 from all NHP species were identified. In western blot, the mAbs displaying the best functionality displayed a more limited cross-reactivity.

Determination of the exact degree of cross-reactivity of antibodies with a protein from different species is highly dependent on how reliable the quantification of the proteins is. Even when comparing the same recombinant HUM protein, obtained from different manufacturers, discordant ELISA results can be obtained although the proteins should yield identical standard curves (Aziz et al., 1999; Bienvenu et al., 1993). Hence, comparison of HUM and NHP cytokines in a HUM cytokine ELISA may reflect different accuracies in how well the cytokines have been quantified as much as a true difference in cross-reactivity, in particular if the cytokines are obtained from different sources. In this study, cytokines were semi-quantified in Western blot using a tag-specific mAb and, based on concentrations defined in this manner, the optimal single mAb reagents and capture assays defined displayed a high degree of cross-reactivity with Old World and New World NHP.

Assessment of panels of mAbs for NHP cross-reactivity obviously presents an advantage compared to evaluating established single mAb reagents pre-selected solely based on reactivity with HUM cytokines. This aspect is even more relevant when identifying NHP-reactive capture assays since both the capture and detection mAb in the pair, by chance, have to display a high degree of cross-reactivity. Several studies illustrate the difficulties associated with identifying capture assay mAb pairs selected for HUM cytokines/factors that cross-react well with NHP. When Luminex multiplex kits for HUM cytokines from five commercial vendors were assessed for cross-reactivity with IFN-γ and IL-2 from five different Old World NHP, none of the kits displayed good functionality; however, all kits worked well for detection of chimpanzee IFN-γ and IL-2 which are more homologous to the HUM cytokines (Giavedoni, 2005). The capture ELISA systems for IFN-γ and IL-2 defined herein also reacted well with chimpanzee cytokines (data not shown). Moreover, when a panel of mAb-based ELISA and ELISpot reagents for various HUM cytokines, from several manufacturers, was assessed for functionality with SAI PBMC; only two out of 13 different mAb pairs displayed functionality (Riccio et al., 2015). One of the functional mAb pairs defined in the study by Riccio et al., 2015, was the anti-HUM IFN-γ mAbs GZ4/7-B6-1-biotin which has been used in many studies on Old World NHP but display a lower functionality in New World NHP where MT126/7-B6-1-biotin or 1-D1K/7-B6-1-biotin are better systems. 1-D1K/7-B6-1-biotin is an established system for HUM IFN-γ and has previously been shown to work for detection of IFN-γ in New World NHP MAR (Woollard et al., 2008), SAI (Kazanji et al., 2006) and AOT (Perlaza et al., 2008). The system also works for Old World NHP as shown herein but has a lower capacity to detect macaque IFN-γ than GZ4/7-B6-1-biotin (Makitalo et al., 2002) or MT126L/7-B6-1-biotin as shown herein. MT126/7-B6-1-biotin was the system that displayed the best reactivity with IFN-γ from all species included in the study.

Old World NHP species used in research are evolutionary related and mAbs that recognize cytokine from one species are likely to cross-react with other Old World NHP species. Similarly, mAbs that cross-react with a New World NHP species are likely to cross-react with other New World species. Still, in some cases mAbs were completely devoid of reactivity with a single species/genus such as anti-IFN-γ mAb 10 that reacted well with Old World NHP IFN-γ apart from macaque IFN-γ. Macaque IFN-γ only differs at one amino acid residue (Ser 103) from for example AGM IFN-γ but that difference is obviously critical for the binding of mAb 10. In a similar manner did mAbs 249, 13D9 and MT2A91 display high reactivity with all NHP IL-2 except for SM IL-2. SM IL-2 differs only at one residue (Arg 5) from RHE/PTM IL-2 and thus a crucial residue in the epitope recognized by these mAbs is identified. When all IL-2 mAbs were assessed for functionality in all possible capture and detection combinations, the mAbs could be grouped in three major epitope clusters depending on which other mAbs they could be combined with (data not shown). Notably, one of the epitope clusters comprised the three mAbs failing to recognize SM IL-2; this group of mAbs also displayed the highest reactivity in Western blot with all NHP species except SM. The fact that subtle differences in the amino acid sequence can render a mAb non-reactive with a single NHP genus emphasizes the need to evaluate all mAbs and capture assays empirically even though cross-reactivity with several other related genera has been shown.

Cytokine sequences can also differ between species within a NHP genus which calls for caution when working with a genus where multiple species are used in research. For example, CYN, PTM and RHE macaques have identical IFN-γ sequences (Tatsumi and Sata, 1997; Villinger et al., 1995) whereas IL-2 is identical in PTM and RHE but differs at two residues in CYN (www.uniprot.org). In Aotus, the situation is reversed with IL-2 being conserved in Ma’s night monkey (AOT), Black-headed night monkey (*A. nigriceps*), Gray-bellied night monkey (*A. lemurinus*) and Spix’s owl monkey (*A. vociferans*) whereas the IFN-γ sequence differs between species in several residues (Hernandez et al., 2002). When the different Aotus IL-2 genes were cloned and sequenced, the IL-2 gene for hamadryas baboon (*P. hamadryas*) was cloned and sequenced in parallel and was found to be identical to all Aotus IL-2 (Hernandez et al., 2002) which is likely due to a PCR contamination since other Old World NHP IL-2 sequences later have been shown to differ from New World NHP IL-2 at several amino acid residues.

With regard to the amino acid sequence used for the recombinant IFN-γ from AOT, it lacked the twelve C-terminal residues and instead the last twelve amino acids from the other two New World NHP, MAR and SAI, that have identical sequences in this region, were used. With the exception of the last 12 amino acids that are not known in AOT, the New World NHP (AOT, SAI and MAR) have very similar IFN-γ sequences and differ only at one amino acid between each other; hence the last 12 amino acids are likely to be similar in AOT IFN-γ too. Also, epitope mapping of several mAbs evaluated in this study revealed that the mAbs that displayed high reactivity with AOT (e.g. MT126L, 1D1K, 7-B6-1) did not involve the C terminus; only mAb G23 recognized an epitope involving the C terminus (Zuber et al., 2016). Hence, the functionality of mAbs/assays to IFN-γ from AOT defined herein is likely to be accurate.

In some cases, the individual reactivity pattern of mAbs with IFN-γ from different species, defined by ELISA by using an anti-IFN-γ mAb for capture and a mAb against the peptide tag on the recombinant cytokines (BAM) for detection, did not match their reactivity pattern in other assays. The most striking example was mAb 111W that displayed equal reactivity with all species when used for capture in ELISA but only reacted with Old World NHP in western blot. Also, GZ-4 displayed equal reactivity with IFN-γ from all species when used as capture mAb in ELISA together with an anti-tag mAb for detection but a preferential reactivity with Old World NHP IFN-γ when mAb 7-B6-1-biotin was used for detection; the lower reactivity with New World NHP should not be caused by mAb 7-B6-1 since that mAb reacted equally well with all species individually as well as when combined with capture mAb MT126L. The dissimilar cross-reactivity patterns observed in different assays could be caused by minor structural alterations in IFN-γ that differ when the antigen is in solution or is bound to a membrane as in the western blot. Potentially, due to the slightly different amino acid sequences of Old and New World IFN-g, the impact of such alterations on mAb binding may differ between IFN-γ from Old *versus* New World NHP. In contrast, the specificity pattern of individual mAbs defined in ELISA in the same manner for IL-2, matched the results seen in other assays. Still, the partly discordant results observed with IFN-γ emphasize the relevance of assessing functionality in all assays and all NHP species intended to be used.

In conclusion, identification of anti-human cytokine mAbs that cross-react with NHP cytokines at an early stage of the mAb evaluation process increases the chance of finding functional mAbs and mAb pairs for various assay applications and for various NHP species. A thorough evaluation of mAbs and assays in different NHP species facilitates the development of reliable reagents for future studies. The identification of mAb reagents and mAb-based capture assays broadly reactive with multiple NHP species is also advantageous for the inclusion in multiplex assays where otherwise restricted NHP reactivity will be a drawback when combining several different anti-cytokine mAb pairs.

## Figures and Tables

**Table 1:** Monoclonal antibodies against human IFN-γ and IL-2 evaluated for cross-reactivity with non-human primates.

MAbs to human IFN-γ		MAbs to human IL-2	
Clone name	Species, IgG subclass^a^	Clone name	Species, IgG subclass
MT126L	rIgG2a	MT2C95	mIgG1
7-B6-1	mIgG1	8-38	rIgG1
GZ4	mIgG1	MT8G10	mIgG1
1-D1K	mIgG1	313-3	mIgG1
111-W	rIgG2a	MT2A91	mIgG2b
124-i	rIgG1	13D9	mIgG2b
118-O	rIgG1	249.9	mIgG1
129-S	rIgG2a	17H12	rIgG2a
109-A	rIgG2b	9.8	rIgG2a
10	mIgG1		
35-D	mIgG1	**Isotype controls**	
G23	mIgG1	**Clone name**	**Species, IgG subclass**
		Ly128	mIgG1
		LDL-18	mIgG2b
		MG1	rIgG1
		MG2	rIgG2a
		16E3	rIgG2b

a All monoclonal antibodies are of IgG isotype from either mouse (m) or rat (r) and have kappa light chains.

## Abbreviations

AIDS: acquired immunodeficiency virus
AGM: African Green monkey
AOT: Ma’s night monkey
BAB: Baboon
CMV: Cytomegalo virus
CYN: cynomolgus macaque
FCS: Fetal calf serum
GFP: green fluorescent protein
HUM: Human
IFN: Interferon
IL: Interleukin
mAb: Monoclonal antibody
MAR: common marmoset
NHP: Non-human primates
PBMC: Peripheral blood mononuclear cells
PBS: Phosphate-buffered saline
PTM: pigtail macaque
RHE: Rhesus macaque
RT: Room temperature
SAI: Squirrel monkey
SIV: Simian immunodeficiency virus
SM: Sooty mangabey
